# Neural networks applied to 12-lead electrocardiograms predict body mass index, visceral adiposity and concurrent cardiometabolic ill-health

**DOI:** 10.1016/j.cvdhj.2021.10.003

**Published:** 2021-10-13

**Authors:** Xinyang Li, Kiran Haresh Kumar Patel, Lin Sun, Nicholas S. Peters, Fu Siong Ng

**Affiliations:** National Heart and Lung Institute (NHLI), Imperial College London, London, United Kingdom

**Keywords:** Adiposity, Cardiometabolic, Neural networks, Obesity, 12-Lead ECG

## Abstract

**Background:**

Obesity is associated with electrophysiological remodeling, which manifests as detectable changes on the surface electrocardiogram (ECG).

**Objective:**

To develop neural networks (NN) to predict body mass index (BMI) from ECGs and test the hypothesis that discrepancies between NN-predicted BMI and measured BMI are indicative of underlying adiposity and/or concurrent cardiometabolic ill-health.

**Methods:**

NN models were developed using 36,856 12-lead resting ECGs from the UK Biobank. Two architectures were developed for continuous and categorical BMI estimation (normal weight [BMI <25 kg/m^2^] vs overweight/obese [BMI ≥25 kg/m^2^]). Models for male and female participants were trained and tested separately. For each sex, data were randomly divided into 4 folds, and models were evaluated in a leave-1-fold-out manner.

**Results:**

ECGs were available for 17,807 male and 19,049 female participants (mean ages: 61 ± 7 and 63 ± 8 years; mean BMI 26 ± 5 kg/m^2^ and 27 ± 4 kg/m^2^, respectively). NN models detected overweight/obese individuals with average accuracies of 75% and 73% for male and female subjects, respectively. The magnitudes of difference between NN-predicted BMI and actual BMI were significantly correlated with visceral adipose tissue volumes. Concurrent hypertension, diabetes, dyslipidemia, and/or coronary heart disease explained false-positive classifications (ie, calculated BMI <25 kg/m^2^ misclassified as ≥25 kg/m^2^ by NN model, *P* < .001).

**Conclusion:**

NN models applied to 12-lead ECGs predict BMI with a reasonable degree of accuracy. Discrepancies between NN-predicted and calculated BMI may be indicative of underlying visceral adiposity and concomitant cardiometabolic perturbation, which could be used to identify individuals at risk of cardiometabolic disease.

## Graphical abstract

**Figure:**
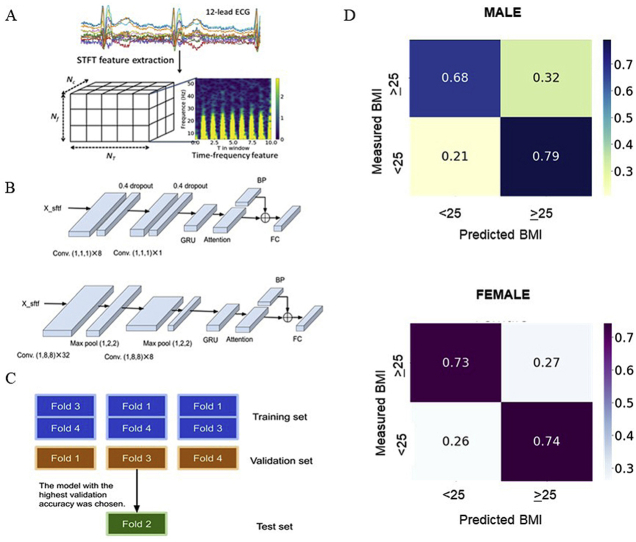

## Introduction

Obesity represents an increasingly common global health problem, with the prevalence of obesity tripling globally in the last 40 years.^1^ Obesity is a recognized cardiovascular risk factor that often coexists with dyslipidemia, insulin resistance, and/or hypertension, the combination of which is referred to as metabolic syndrome.^2^

Body mass index (BMI) is the most common metric to categorize obesity. However, it is an insensitive index of visceral adiposity and does not accurately reflect the presence of underlying metabolic ill-health,^3^ and thus provides an inaccurate measure of cardiometabolic risk. On the other hand, body composition profiling using cross-sectional imaging provides more accurate phenotypic characterization of adipose distribution than standard anthropometric measurements such as BMI, and better discrimination of cardiometabolic risk,^4^ though its utility is limited by practicality and availability.

Obesity has been shown to be associated with a number of electrophysiological changes, owing in part to the direct effect of epicardial adipose tissue on the ionic currents that regulate the cardiac action potential,^5^ and these combined effects manifest as discernible changes on the 12-lead electrocardiogram (ECG), such as QT prolongation.^6^

In this study, we hypothesized that neural networks (NN) can be trained to estimate BMI from the ECG, and importantly, any discrepancies between NN-estimated BMI and measured BMI may provide important information about underlying visceral adiposity and concurrent metabolic ill-health. We trained NNs to predict BMI using ECGs from the UK Biobank and investigated the reasons for differences between NN-estimated BMI and measured BMI.

## Methods

Main methods are outlined below. Further details are provided in Supplemental Materials.

### Data sources and study population

Ten-second resting 12-lead ECGs from 36,856 adult participants recorded in the first imaging visit of the UK Biobank study were used in our analysis.^7^ Magnetic resonance imaging (MRI) scans that were performed in the same instance were also included in our analysis. Details are provided in the Supplemental Methods.

### ECG classification using neural networks

Two forms of ECG input were adopted: the full 10-second trace and averaged single beat. Single beats were obtained using QRS detection from full ECG traces, truncated into the same lengths of 1 second, aligned with R peak at the center of the beat segment. Averaged single beat was calculated thereafter. For both forms of the input, short-time Fourier transforms (STFT) were applied to extract temporal and frequency features. For the full trace, the window length and shift of STFT were 0.5 second and 0.1 second, respectively. For the averaged single beat, since the beat length is shorter, shorter window lengths and shifts were used: window length and shift were 0.2 and 0.01 second, respectively.

**Figure 1:**
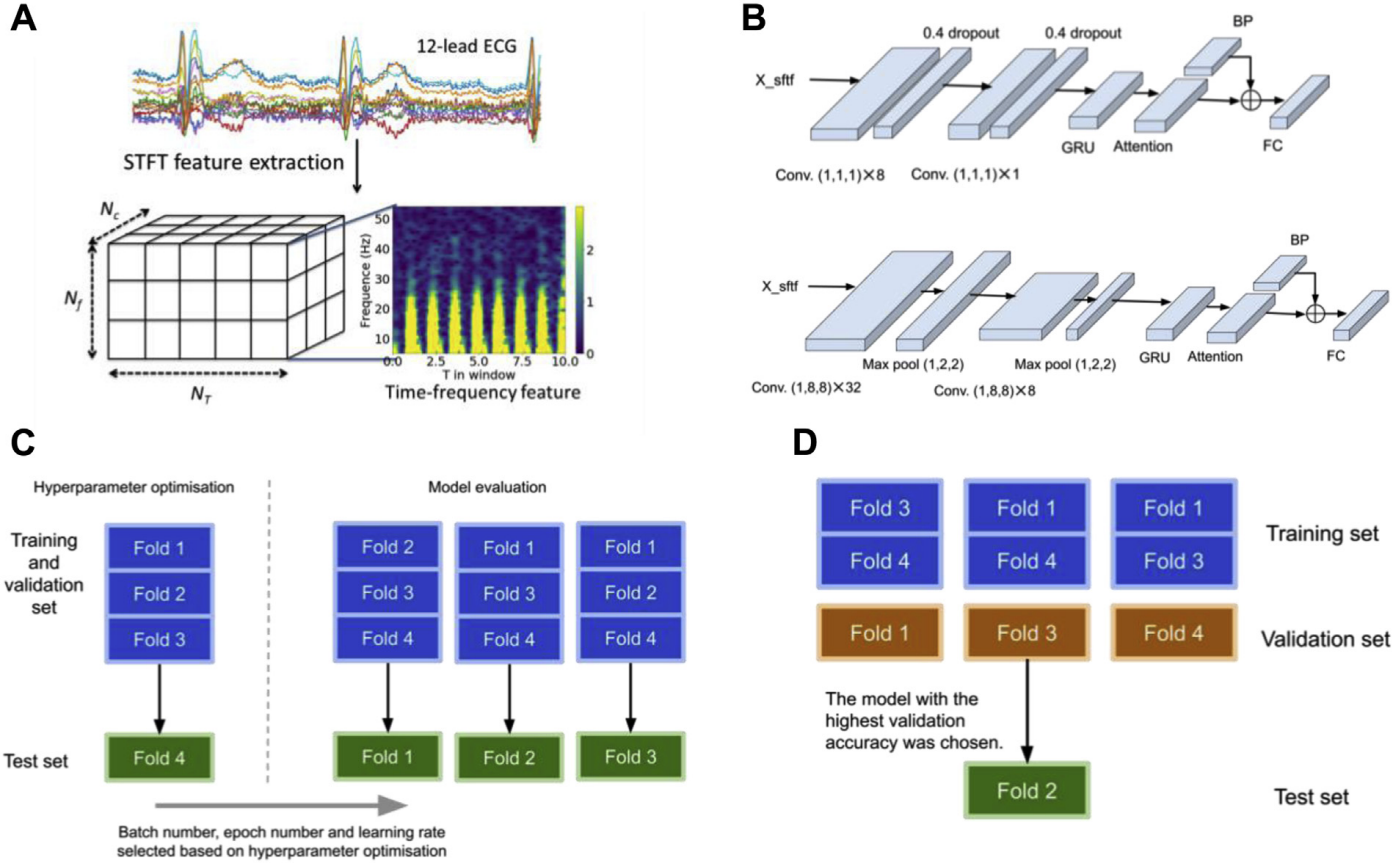
Data preprocessing and model architectures. A: Data preprocessing with short-time Fourier transforms (STFT). **B:** The architectures of the 2 proposed models: both models adopt convolution layers (conv.), gated recurrent units (GRU), and attention layers. Compared to model 1, model 2 has more convolution layers. The 2 architectures are used for categorical classification of obesity by body mass index (BMI) and for BMI as a continuous variable. **C:** The 2 stages of model selection after data were divided into 4 folds. Firstly, there was hyperparameter optimization using the fourth fold as the test set. Secondly, with the selected model architecture and hyperparameters, the remaining 3 split settings were used to generate final results. **D:** Within each split setting, different combinations of training and validation folds were tested in a rotational manner with the test fold fixed. In the illustrated example, fold 2 was used as the test data. Therefore, for each test fold, 3 models were trained and the model with the highest validation accuracy was chosen to be tested by the test set.

The frequency spectrum of QRS complexes is usually within a range of ∼8–50 Hz, and that of T and P waves within ranges of ∼0–10 Hz and 5∼30 Hz, respectively. Thus, STFT components corresponding to 5–55 Hz were used as input for the networks to encapsulate these ECG components. The STFT preprocessing with full traces is illustrated in Figure 1A. After STFT, inputs were transformed into a tensor (*X*) with shape N_T_-by-N_f_-by-N_c_, where N_T_ = 100 (number of windows), N_f_ = 50 (number of frequency components), and N_c_ = 12 (number of leads) for the full trace, and N_T_ = 100, N_f_ = 10, and N_c_ = 12 for the average beat.

Figure 1B shows the architectures of the 2 proposed models: both models adopt convolution layers (conv.), gated recurrent units (GRU), and attention layers.^8^^,^^9^ Compared to model 1, model 2 is more complex, with more convolution layers. The GRU output was concatenated with band-power features, and then fed forward into the final fully connected (FC) layer. Band power features are equivalent to the sum of STFT elements/features of the corresponding frequency components over all the windows. The advantage of using band power features before the FC layer is that it serves as a shortcut similar to a deep residual network.^10^ The learning of the more complicated parts of the network, ie, GRU, would be skipped if the “shortcut” proved to be more useful. In this work, band power features from 3 overlapping bands—10∼25, 20∼35, and 30∼50 Hz—were used. These yielded 36 (12 leads × 3) band power features.

Both architectures were tested with both categorical classification and regression tasks. In the categorical classification task, the final FC layer fed into a softmax layer to obtain the probability estimate class label *y*_*i*_ of the subject *i*, with input data *X*_*i*_ being the overweight/obese class—ie, *p*(*y*_*i*_ = 1 | *X*_*i*_). Class weights were used to balance the training dataset. In the regression model, the continuous BMI estimates from the FC layer was the final output.

### Cross-validation

For each sex, the data were randomly split into 4 folds for leave-1-fold-out cross-validation. For every model trained, 2 out of 4 folds were used as the training set, 1 as the validation set, and 1 as the test set. Before a test set is applied to a trained model, all 3 other folds would have rotated to be the validation set in 1 of the 3 models trained by the remaining 2 training sets. The model yielding the highest validation accuracy was then selected to be tested by the test fold. Each fold when used as a test set is referred to as a unique split setting. The cross-validation process can further be divided into network hyperparameter optimization and subsequent model evaluation. The split setting for hyperparameter optimization used the fourth fold as the test set, as shown in Figure 1C and Supplemental Methods. Further details of the network hyperparameter optimization can be found in the Supplemental Methods. With the best-performing hyperparameters, further models were trained with folds 1–3 as test sets. Again within each of these split settings, different combinations of training and validation folds were tested in a rotational manner, with the test fold fixed, as shown in Figure 1D. Therefore for each model, 50% of the total data were used for training, 25% for validation and 25% for testing. In this way, the estimation results for subjects of 1 fold were obtained by models trained and validated by subjects from different folds. Finally for out-of-bag validation, we randomly selected 1 model generated from the model evaluation stage and tested the accuracy of the model on a set of 2278 ECG samples that were not previously seen by the model at any stage (Supplemental Methods).

### Network hyperparameter optimization

Network hyperparameter optimization is described in the Supplemental Methods.

### Statistical analysis

As BMI is a surrogate measure of adiposity, we further investigated the association between visceral adipose tissue (VAT) volumes and BMI estimation using NNs. We conducted linear regression analysis to predict VAT volumes using △BMI, ie, the difference between NN-predicted and true BMI (calculated from weight and height), with the latter as the control variable.

To determine if BMI estimation was affected by the presence of cardiometabolic comorbidities, the associations of NN BMI estimates with 4 comorbidities—hypertension, coronary heart disease (CHD), diabetes, and dyslipidemia—were examined using logistic regression adjusted for age and measured BMI. Details of the International Classification of Diseases (ICD) codes can be found in Supplemental Table 1.

χ^2^ tests with multicomparison correction (Benjamini-Hochberg) were also conducted to determine whether the frequencies of these comorbidities were significantly different between 4 different estimation groups in the binary classification, ie, true-positive (TP) vs false-negative (FN) and true-negative (TN) vs false-positive (FP) groups. *P* < .05 was considered as significant in all analyses.

## Results

### Study population

A total of 36,856 ECG samples were available with BMI information as labels (19,049 female, 52%). The mean ages for male and female subjects were 63 ± 8 years and 61 ± 7 years, respectively. The mean BMI for male and female subjects were 26 ± 5 kg/m^2^ and 27 ± 4 kg/m^2^, respectively. A total of 68% of male subjects and 53% of female subjects in our study cohort were labeled as overweight or obese (BMI ≥25 kg/m^2^).

### Hyperparameter optimization

Table 1 summarizes the binary classification (normal weight vs overweight/obese) accuracies for hyperparameter optimization and model selection, using data from the fourth fold for each sex as the test fold. There were minimal differences in the classification accuracies among different settings. The results are robust against different batch sizes, number of training epochs, and learning rates. Model 2 contained more convolutional layers but did not outperform model 1. For comparison, logistic regression was also implemented using maximum absolute values of raw ECGs as the input. NN models outperformed logistic regression at differentiating normal weight vs overweight/obese. Given the results in Table 1, parameters and the architecture in setting 4 were adopted for the further analyses, and data from the fourth folds of both sexes were excluded from the test sets in the following sections.

**Table 1:** Binary classification (body mass index ≥25 vs <25) results with different models

Setting no.	Model	Input data	Batch size	N epoch	Learning rate	Accuracy (%)	
						Female	Male
1	Model 1	Averaged single beat	32	50	0.001	72.06	75.01
2	Model 1	Full trace	32	50	0.001	72.21	75.37
3	Model 1	Averaged single beat	50	100	0.005	71.30	74.76
4	Model 1	Full trace	50	100	0.005	72.84	75.19
5	Model 2	Averaged single beat	50	200	0.005	71.17	75.28
6	Simple logistic regression using max absolute values of raw ECGs					60.42	62.10

The fourth fold was used as the test set to test sensitivity against data input format, model architecture, and training settings (ie, Setting no. 1-6). The results are generally robust against the variations of settings. The more complex model 2 did not outperform model 1, and simple logistic regression was inferior to neural network models.

ECGs = electrocardiograms.

### BMI classification using NNs applied to ECGs

After excluding data from the fourth fold, the numbers of samples for model evaluation were 14,299 and 13,372 for female and male subjects, respectively. The baseline characteristics are detailed in Supplemental Table 2. After excluding data from the fourth fold, VAT volumes were available for 2,724 male and 2,966 female subjects.

**Figure 2:**
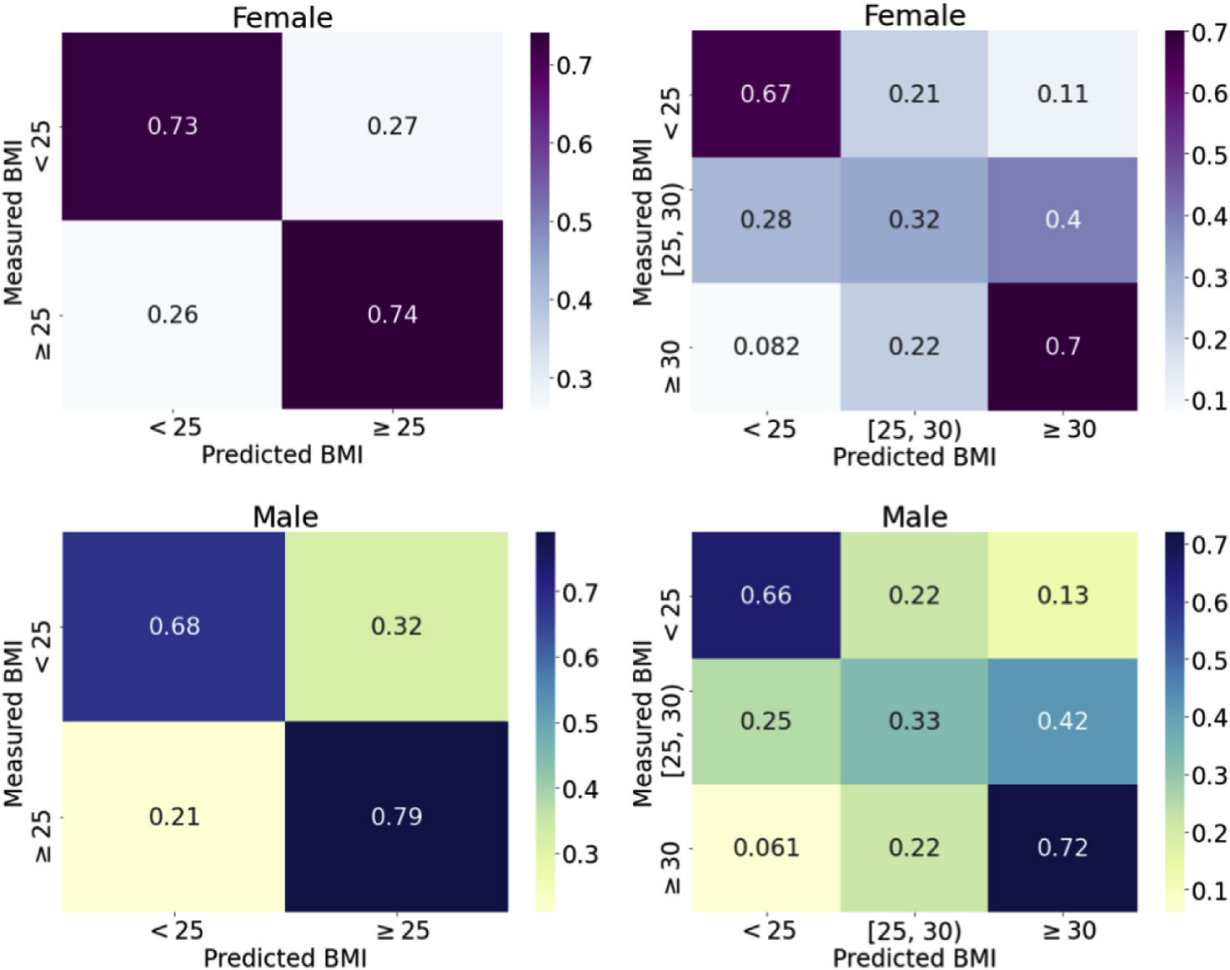
Confusion matrices of binary classification (BMI ≥25 kg/m^2^) and 3-class classification (BMI <25 kg/m^2^, 25 kg/m^2^ ≤ BMI < 30 kg/m^2^, BMI ≥30 kg/m^2^): For the binary classification, the accuracies are 75% and 73% for male and female subjects, respectively. For the 3-class classification, the accuracies are 51% and 56% for male and female subjects, respectively. BMI = body mass index.

The confusion matrices corresponding to binary classification, ie, normal weight vs overweight/obese, and 3-class classification, ie, normal weight (BMI <25) vs overweight (BMI 25–30) vs obese (BMI ≥30), are presented in Figure 2. For the binary classification, the proposed NN model achieved accuracies of 75% and 73% in male and female subjects during the model evaluation phase, respectively, and for the 3-class classification, the accuracies are 51% and 56% for male and female subjects, respectively. In the three-class classification, there are relatively fewer misclassifications between normal-weight and obese groups, with 13% obese and 6% normal-weight male subjects and 11% obese and 8% normal-weight female subjects being misclassified. Similar accuracies were achieved during out-of-bag testing (Supplemental Figure 1).

### Continuous BMI and VAT estimation using NNs applied to ECGs

**Figure 3:**
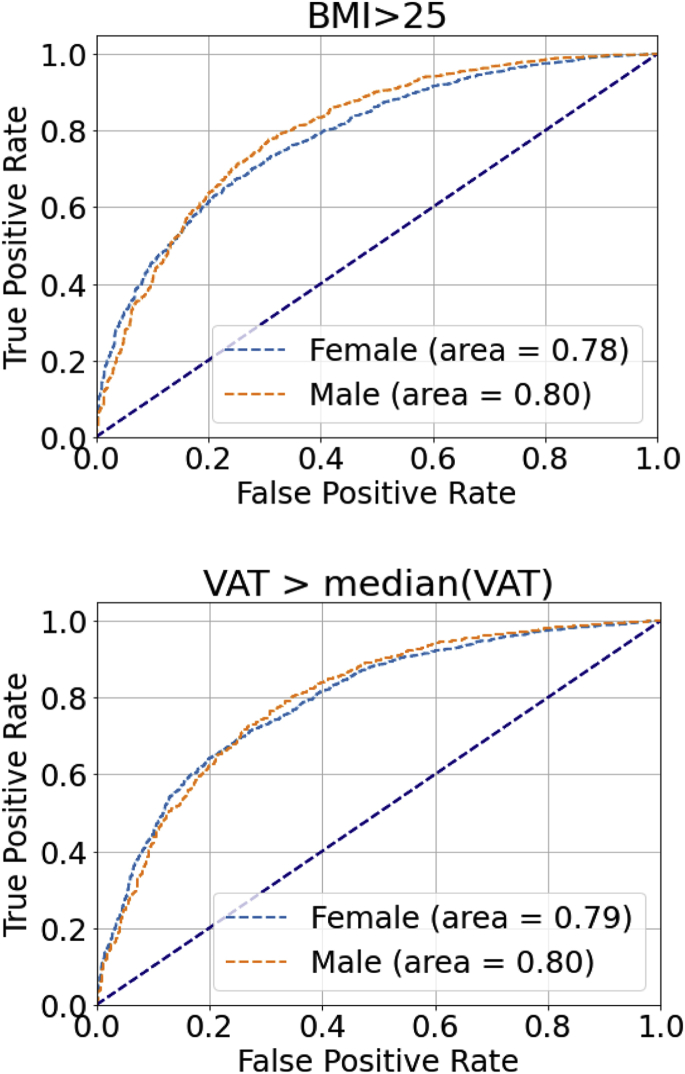
The receiver operating characteristic curves for binarized body mass index (BMI) and visceral adipose tissue (VAT) estimation. Continuous neural network estimation regression models were fitted for both BMI and VAT. The estimated BMI and VAT were used to predict actual BMI < or ≥ 25 kg/m^2^ (ie, normal weight vs overweight/obese) and actual VAT > or < median actual VAT, respectively, to show the performance of the regression models. For BMI, the areas under curve (AUC) were 0.80 and 0.78 for males and females, respectively. As there is no established definition of obesity based on VAT, median VAT for each sex was used; the AUC were 0.80 and 0.79 for males and females, respectively.

The regression NN model was applied for both continuous BMI and VAT estimation. For continuous BMI estimation, the root-mean-square error of male and female participants was 3.2 kg/m^2^ and 4.0 kg/m^2^, respectively. We also tested binarizing the continuous BMI estimates to classify normal weight vs overweight/obese. The receiver operating characteristic curve is presented in Figure 3. The areas under curve (AUC) for male and female participants were 0.80 and 0.78, respectively.

For continuous VAT estimation, the root-mean-square errors of male and female subjects were 1.9 L and 1.2 L. To further analyze the performance of the regression model in estimating VAT from ECGs, we binarized our sample into 2 groups based on the median measured VAT value. We determined the ability of the NN to predict whether VAT volume is larger or smaller than the median VAT volume. The median VAT is used in this binarization because there is no conventional definition of obesity based on VAT. The AUC were 0.80 and 0.79 for male and female subjects, respectively.

### Visceral adipose tissue volume accounts for discrepancies between measured and NN-derived BMI

After excluding data from the fourth fold, samples with VAT volumes were available for 2,966 female and 2,724 male subjects. Table 2 summarizes the results of the linear regression analysis between VAT volumes (L) and △BMI (the difference between NN-predicted and true BMI), adjusted for true BMI. The correlations between VAT volume and △BMI were significant in both sexes, suggesting VAT volume increases with increasing discrepancy between the NN-predicted BMI and true BMI. For every unit increment in △BMI, VAT increases by 0.23 L/(kg/m^2^) (CI 0.19–0.26 L/[kg/m^2^]) and 0.11 L/(kg/m^2^) (CI 0.10–0.13 L/[kg/m^2^]) in male and female subjects, respectively.

**Table 2:** The association between ΔBMI and visceral adipose tissue volume

	N	β (per unit ΔBMI)	β (per SD ΔBMI)	*P*	R^2^
Female	2966	0.11 [0.10–0.13]	0.45 (SD = 3.93) [0.38–0.51]	<.001	0.61
Male	2724	0.23 [0.19–0.26]	0.73 (SD = 3.81) [0.62–0.83]	<.001	0.62

Linear regression modeling of VAT volumes (L) with ΔBMI (kg/m^2^) adjusted for true BMI show that VAT increases with every unit increment in ΔBMI: 0.11 L/(kg/m^2^) and 0.23 L/(kg/m^2^) in females and males, respectively. Beta values per standard deviation (SD) are also shown.

BMI = body mass index; ΔBMI = difference between neural network–predicted and true BMI; VAT = visceral adipose tissue.

### NN-estimated BMI and VAT are associated with cardiometabolic diagnoses

**Figure 4:**
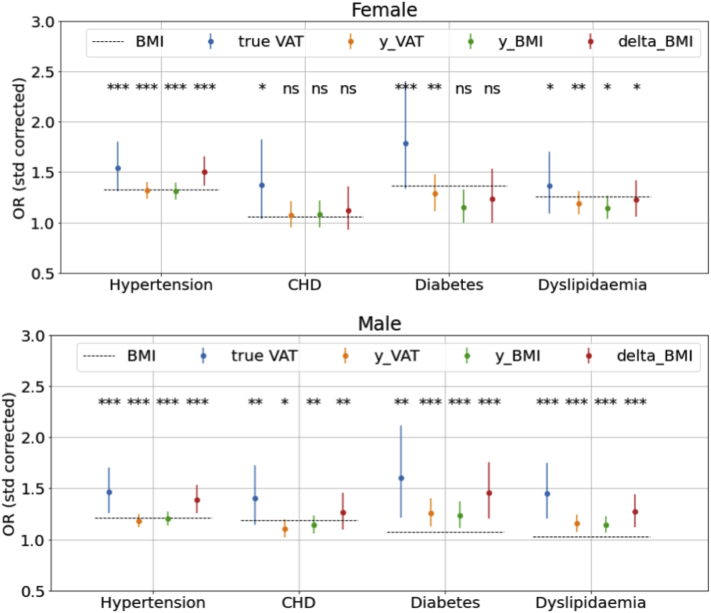
Neural network (NN)-predicted visceral adipose tissue (VAT) and body mass index (BMI) are associated with increasing odds of cardiometabolic comorbidities. Odds ratio (OR) values corrected by standard deviation (std) and associated confidence intervals corresponding to different measures, true VAT, y_VAT (NN-predicted VAT), y_BMI (NN-predicted BMI), and delta_BMI (ΔBMI, difference between NN-predicted and measured BMI), are presented. The OR for true BMI is presented in dashed lines. For example, with every standard deviation increment in △BMI, the risks of having hypertension are 1.4- and 1.5-fold greater in males and females, respectively. CHD = coronary heart disease. ∗*P* < .05, ∗∗*P* < .01, ∗∗∗*P* < .001.

Figure 4 presents multivariable logistic regression results showing associations of 4 obesity indices—true measured VAT, NN-predicted VAT (y_VAT), NN-predicted BMI (y_BMI), and △BMI (NN-predicted BMI − measured BMI)—with 4 comorbidities, ie, hypertension, CHD, diabetes, and dyslipidemia, while adjusting for age and measured BMI. True measured VAT is also presented for comparison. We found that risk of hypertension and dyslipidemia increases with increasing y_VAT, y_BMI, and △BMI in female subjects. We also observed statistically significant increment in risk of all 4 comorbidities with increasing y_VAT, y_BMI, and △BMI in male subjects. The odds ratio (OR) of having hypertension with every standard deviation increment in △BMI is similar to that of measured VAT—1.4- and 1.5-fold-greater in males and females, respectively. In male subjects, the ORs of all 4 comorbidities are consistently greater for △BMI than true BMI while also being closer to the OR found in measured VAT. Therefore, the difference between NN-predicted BMI and true BMI (△BMI) is likely a better predictor of cardiometabolic diagnoses than true measured BMI.

### Concurrent cardiometabolic diagnoses explain NN-derived overestimation/underestimation of BMI

**Figure 5:**
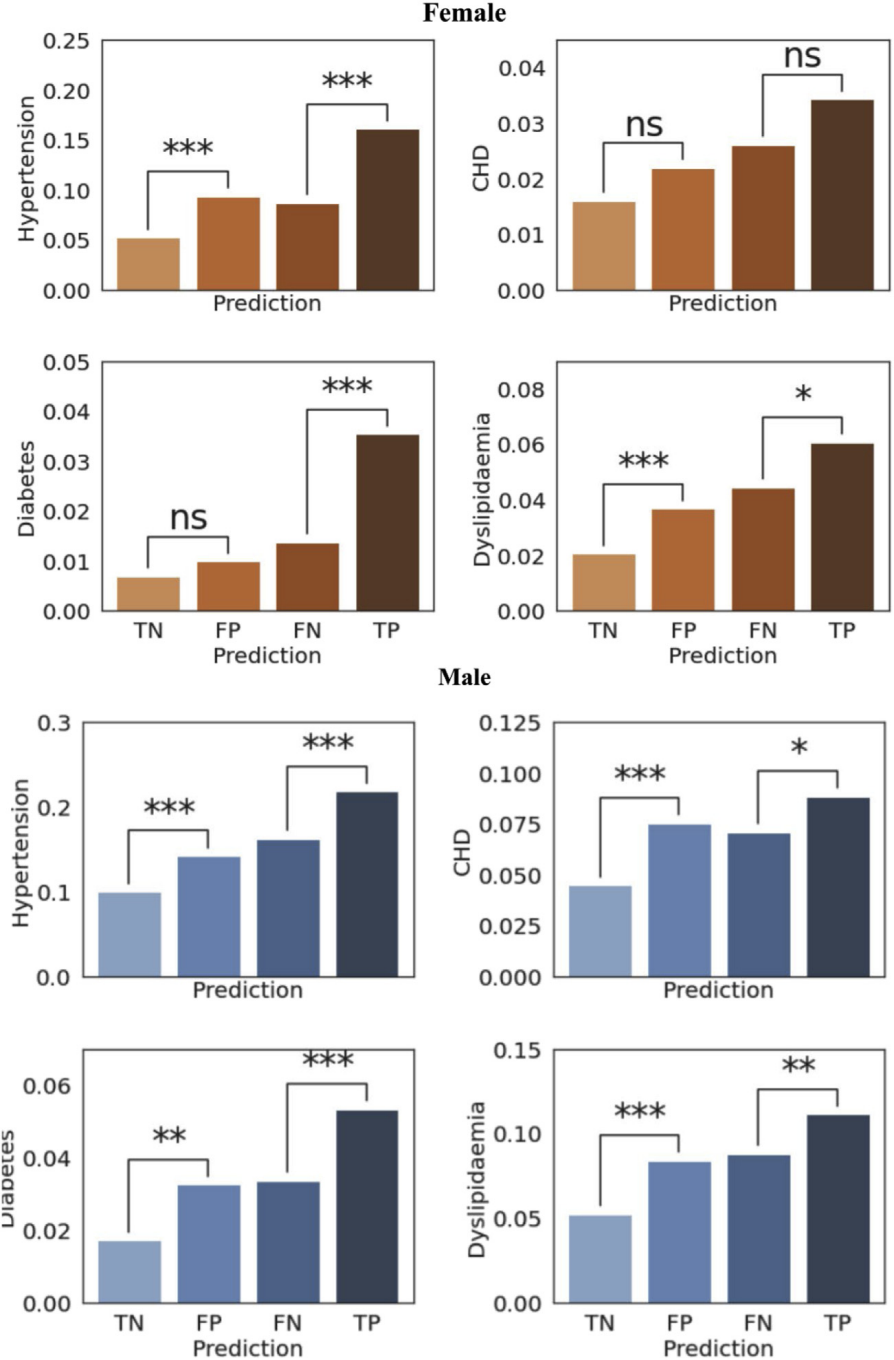
Concurrent cardiometabolic diagnoses explain neural network over-/underestimation of body mass index. Prevalences of the 4 conditions are presented for the 4 prediction groups: true-negative (TN), n = 4926/2902 (female/male); false-positive (FP), n = 1824/1380 (female/male); false-negative (FN), n = 1956/1865 (female/male); true-positive (TP), n = 5593/7225 (female/male). There was higher prevalence of hypertension and dyslipidemia in the FP group compared to TN, suggesting that normal-weight participants with cardiometabolic comorbidities may share similar ECG features with overweight/obese participants, leading to those normal-weight individuals being misclassified as overweight. Conversely, the prevalence of hypertension, diabetes, and dyslipidemia in the FN group is lower than that in the TP group, suggesting that overweight/obese participants without cardiometabolic comorbidities, ie, relatively healthy overweight/obese, may have electrocardiogram features similar to those recorded in normal-weight participants, leading to their being misclassified as normal-weight. ∗*P* < .05, ∗∗*P* < .01, ∗∗∗*P* < .001.

Comparison of comorbidities between different NN categorical estimation groups, ie, TP (BMI ≥25 correctly classified) vs FN (BMI ≥25 but misclassified as <25), and TN (BMI <25 correctly classified) vs FP (BMI <25 but misclassified as ≥25) groups, is shown in Figure 5. In female subjects, the prevalence of hypertension and dyslipidemia was greater in the FP group, where the BMI was overestimated by the NN, compared to TN. In male subjects, the prevalence of all 4 comorbidities was greater in the FP group compared to TN, suggesting that normal-weight participants (BMI <25 kg/m^2^) are more likely to be identified as overweight/obese by the NN if they have these comorbidities. In other words, the ECGs recorded from normal-weight participants with concurrent cardiometabolic ill-health may share characteristics of ECGs recorded from overweight/obese participants. A reverse pattern of misclassification was also observed when comparing the FN and TP groups, whereby overweight individuals were more likely to be misclassified as normal-weight by the NN if they did not have concomitant cardiometabolic comorbidities: in females, prevalence of hypertension, diabetes, and dyslipidemia was lower in the FN group compared to the TP group; in males, the prevalence of all 4 comorbidities was lower in the FN group compared to the TP group.

## Discussion

Using data from the UK Biobank, we adopted NN models to classify individuals as normal weight (BMI <25 kg/m^2^) or overweight/obese (BMI ≥25 kg/m^2^) using the 12-lead ECG, achieving accuracies of 73% and 75% in male and female subjects, respectively. The difference between NN-predicted and actual BMI was also found to be correlated with measured VAT volumes. NN-predicted BMI was indicative of cardiometabolic morbidity. Additionally, we demonstrated that participants with calculated BMI <25 kg/m^2^ who had concomitant hypertension, diabetes, dyslipidemia, or CHD were more likely to be identified as overweight/obese by our NN. On the other hand, those with actual BMI ≥25 kg/m^2^ without cardiometabolic perturbation were more likely to be identified as normal weight, ie, BMI <25 kg/m^2^. Our data suggest that NN-derived BMI estimates based on ECGs may provide a better indication of visceral adiposity and underlying cardiometabolic risk than conventional BMI.

### Extracting adiposity-associated changes in ECG by NN

It was recently estimated that more than 1.9 billion adults are overweight (BMI ≥25 kg/m^2^) and more than 650 million of those are obese (BMI ≥30 kg/m^2^).^11^ Obesity is associated with proarrhythmic electrophysiological remodeling that manifests on the 12-lead ECG, which is reversible with weight reduction strategies.^12^ This includes P wave and PR interval^13^ and QRS prolongation^14^ with implications for atrial and ventricular arrhythmogenesis. The proarrhythmic substrate in obesity is likely the result of ionic channel modulation,^15^ myocardial fibrosis,^16^ and connexin downregulation.^17^ Given that obesity influences a range of ECG parameters, we developed a NN that can utilize all possible underlying features of a resting 12-lead ECG to predict BMI.

**Figure 6:**
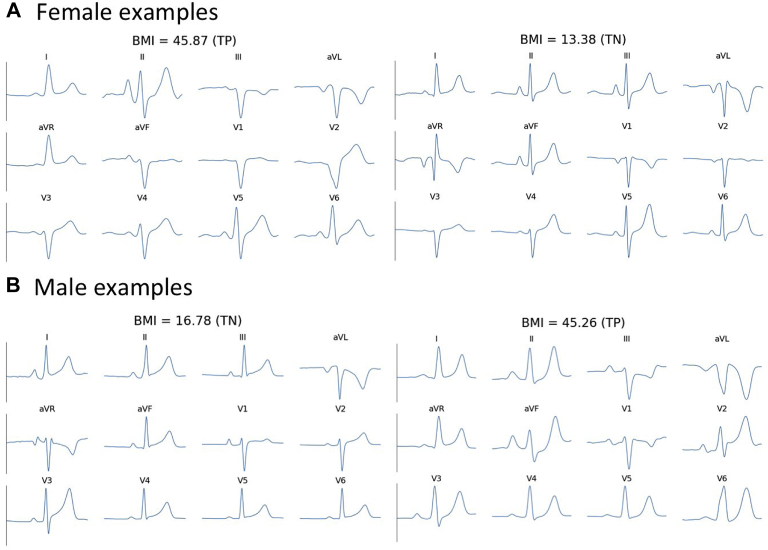
Examples of QRS complexes from different estimation groups. The differences between electrocardiograms from normal-weight (body mass index [BMI] <25 kg/m^2^) and overweight/obese individuals (BMI ≥25 kg/m^2^) are difficult to discern by visual evaluation, though the proposed neural network model captures electrocardiographic changes due to obesity. TN = true-negative; TP = true-positive.

Different NN models, including convolutional and recurrent neural networks, have been applied to ECGs for various classification tasks.^18^^,^^19^ In this work, we proposed architectures consisting of both convolutional layers and recurrent NN units, and we showed that subtle differences in ECG due to obesity can be detected by the NN models. Figure 6 demonstrates that although morphological differences between normal weight and overweight/obese may be difficult to quantify visually, our models can predict BMI ≥25 kg/m^2^ with accuracies >73%, which is comparable to that of routine cardiovascular diagnostic tests.^20^ Models with a greater number of convolutional layers did not improve classification accuracy and classification accuracy was robust against batch size and learning rate.

We also tested simple logistic regression models with maximum absolute amplitudes of raw ECG as inputs. If the differences in ECGs between normal-weight and overweight/obese subjects were attributable to differences in amplitude as a consequence of greater impedance associated with obesity,^21^^,^^22^ it may be possible to differentiate the 2 groups using maximum absolute amplitudes with a simple classifier. However, simpler models utilizing amplitude features alone yield a lower degree of accuracy than the NN, suggesting that amplitude attenuation does not make a significant contribution to NN-derived ECG classification.

### Differences between NN-predicted and actual BMI reflect underlying visceral adiposity

The classification for obesity is commonly based on anthropometric measurements such as BMI. These methods are insensitive to body composition and do not differentiate between muscle mass, adipose tissue, or adipose volumes in different depots. VAT, which constitutes adipose tissue surrounding internal organs, has been shown to be more detrimental to health than subcutaneous fat tissue.^23^ For example, epicardial adiposity has been associated with coronary artery disease^24^ and arrhythmogenesis,^25^ with electrophysiological remodeling most profound in regions adjacent to epicardial adipose tissue.^26^ Despite this, there is no clinically accessible method by which VAT volumes, and thereby the associated arrhythmic risk, can be easily estimated.

Given that BMI is only a surrogate measure of adiposity, we demonstrated that differences between NN-predicted and actual BMI are correlated with VAT volumes. Controlling for actual BMI, the greater the discrepancy between NN-predicted and actual BMI, the greater the VAT volume (Supplemental Figure 2). Despite limited datasets with both VAT volumes from MRI and ECG traces, we showed in Figure 3 the AUC of using NN-estimated VAT to predict true measured VAT with median VAT as cut-off is around 0.80. Nevertheless, we speculate that as data accrue in the UK Biobank, more accurate NN models on 12-lead ECG could be developed to identify lean individuals who have a high VAT volume and consequently a higher risk of cardiometabolic disease. At present, adipose volume can only be accurately determined using time-consuming and expensive modalities such as dual-energy x-ray absorptiometry (DEXA) and MRI. NNs applied to ECG may provide a more convenient low-cost alternative to identify individuals with higher risk of cardiometabolic disease. Although we acknowledge that NN-based derivations are unlikely to replace routine anthropometry, our results demonstrate that they nonetheless provide added value to standard indices.

### NN-derived BMI measures reflect concomitant cardiometabolic morbidity

Obesity is strongly associated with cardiometabolic perturbation.^27^ Just as differences between chronological and biological age have been attributed to concomitant comorbidity,^28^ our logistic regression analysis showed that NN-derived BMI measures, ie, NN-predicted BMI and △BMI, are associated with hypertension and dyslipidemia in females and with hypertension, diabetes, dyslipidemia and CHD in males. By comparing prevalence of these morbidities among normal-weight and overweight/obese groups, we show that some normal-weight participants with concomitant cardiometabolic morbidity had NN-predicted BMIs that misclassified them as overweight/obese. Similarly, some overweight/obese participants were misclassified as having a normal weight in the absence of concomitant comorbidity. Our findings are consistent with studies that have shown over a third of normal-weight individuals may be metabolically unhealthy,^29^ which confers a cardiometabolic risk similar to that associated with obesity.^30^ Importantly, our data suggest that NN-derived BMI estimates based on ECGs may provide a better indication of underlying cardiometabolic risk than BMI.

### Limitations and future work

Although we show NN-predicted BMI is influenced by adiposity, our analysis did not account for differences in intra-depot adipose variation. For instance, the fraction of VAT that constituted epicardial and adipose tissue around other abdominal organs was not known. We anticipate that with more detailed information on body composition it may be possible to provide organ-specific risk profiles using artificial intelligence. Theoretically, it would have been preferable to train NN with VAT volumes as labels. However, because VAT labels were available for only 7,461 ECG samples, the OR of NN-predicted VAT in predicting cardiometabolic perturbation is not superior to NN-predicted BMI. Future work could include developing a VAT-specific model for risk stratification and the application of NN-derived BMI to follow-up data in the UK Biobank to compute risks for development of cardiometabolic ill-health. Similarly, it would be of interest to identify the ECG features utilized by the NN to predict BMI.

## Conclusion

Application of NN to ECGs can determine BMI to a degree of accuracy comparable to that of existing diagnostic tests. The differences between NN-predicted and calculated BMI can partly be attributed to underlying adiposity, particularly of the visceral subtype, and the presence of CHD, diabetes, hypertension, and/or dyslipidemia. NN-derived parameters may be useful in supplementing conventional anthropometric measures of obesity to identify individuals at higher risk of cardiometabolic ill-health.

## Funding Sources

This work was supported by the British Heart Foundation (RG/16/3/32175) and the National Institute for Health Research (NIHR) Imperial Biomedical Research Centre (BRC).

## Disclosures

The authors have no conflicts to disclose.

## Authorship

All authors attest they meet the current ICMJE criteria for authorship.

## Patient Consent

UK Biobank participants provided informed consent for their data to be used for health-related research.

## Ethics Statement

Data used in these analyses were accessed under application number 48666 and are available upon application to the UK Biobank. The UK Biobank study was approved by the North West Multicentre Research Ethics Committee. This study conforms to the Declaration of Helsinki.
