# Supplementary material for: Neural networks applied to 12-lead electrocardiograms predict body mass index, visceral adiposity and concurrent cardiometabolic ill-health
Source: Cardiovasc Digit Health J. 2021 Oct 13;2(6 Suppl):S1–S10. doi: 10.1016/j.cvdhj.2021.10.003 (PMC8669785; doi:10.1016/j.cvdhj.2021.10.003)
Supplement: Supplementary Material [file mmc1.docx]

**Supplementary Materials:** **Neural networks applied to 12-lead electrocardiograms predict body mass index, visceral adiposity and concurrent cardiometabolic ill-health**

**Short title: Adiposity can be detected from ECG by neural networks**

**Authors**: Xinyang Li PhD^a,^*, Kiran Haresh Kumar Patel BSc MRCPa,*, Lin Sun BSc^a^, Nicholas S Peters MD FHRS^a^, Fu Siong Ng PhD FRCP^a^

^a^National Heart and Lung Institute (NHLI), Imperial College London, UK

*joint first authors

**Correspondence to:**

Dr Fu Siong Ng

4th Floor, ICTEM Building,

Imperial College London,

72 Du Cane Road,

W12 0NN, United Kingdom.

E-mail: f.ng@imperial.ac.uk

**Conflict of interest:** The authors have no conflicts to disclose.

**Word count:** 982

## **Supplementary Methods**

## **Data sources and study population**

10-second resting 12-lead ECGs from 36,856 adult participants from the UK Biobank were used in our analysis^1^. Models with 19,049 male and 17,807 female participants were developed separately to obviate sex as a confounding factor. An additional 2,278 ECG samples (n=1,148 male; n=1,130 female) recorded at the first repeat imaging visit were kept aside for final testing of trained model. ECGs were acquired at a sampling rate of 500Hz using the GE Cardiosoft system, with participants lying in a supine position. BMI and ECG were recorded at the same visit, and overweight or obese individuals were defined in a single group with BMI>25kg/m^2^. Abdominal subcutaneous adipose tissue (SAT) and visceral adipose tissue (VAT) volumes (litres, L) for 4,158 males and 4,499 females had previously been derived from magnetic resonance imaging^2^. Comorbidity was defined using primary and secondary International Classification of Disease (ICD)-10 summary diagnoses and listed in **Supplementary Table 1**. Baseline characteristics are shown in **Supplementary Table 2**.

## **Model development**

The proposed models were implemented using Tensorflow (Google, Mountain View, CA) and Python 3.6.

**Network hyperparameter optimisation**

The hyperparameters were determined by the best performing validation results. Dropout layers with dropout rate of 0.5 were added to the models to avoid overfitting^6^. The Adam optimiser with binary cross-entropy and mean-squared-error (MSE) was adopted for the categorical and continuous regression BMI classifications respectively^7^. For each sex, different learning rates and batch sizes were tested with data from the fourth fold as the test fold. The data from the fourth fold was excluded for further test result evaluation.

To validate the robustness of the model we tested our algorithm on an additional 2,278 ECG samples (n=1,130; female 49%) recorded at the first repeat imaging visit. These samples have never been seen by any model, nor included in the training or hyper-parameter tuning. The labels for this new ECG subset are the BMI measured at the repeat imaging visit. We randomly selected one model generated during the model evaluation stage for validation. The NN model achieved accuracies of 76% and 72% in males and females, respectively, and for the three-class classification, the accuracies were 54% and 52% for males and females, respectively. These results are similar to the accuracies detailed in the main manuscript **(Supplementary Figure 1).**

**Supplementary Results**

**The discrepancy between NN-predicted and actual BMI (ΔBMI)** **is indicative of VAT volume**

To demonstrate the association between VAT and the discrepancy between NN-estimated BMI and actual BMI (ΔBMI), we constructed the following regression model:

**VAT ~ 𝛽_0_ *BMI +*β** ΔBMI + const.**

where 𝛽_0_ and *β* are the regression coefficients corresponding to actual BMI and ΔBMI, respectively. To illustrate the association, **ΔVAT** was defined as shown below, by rearranging the equation above. **ΔVAT** gives the portion of VAT explained by ΔBMI.

**ΔVAT:=VAT - 𝛽_0_ *BMI - const.**

**Supplementary Figure 2** shows the portion of VAT (**ΔVAT)** that is explained by ΔBMI.

**Supplementary Table 1:** Definitions of comorbidity

| **Comorbidity** | Code | Definition |  |
| --- | --- | --- | --- |
| **Hypertension** | I10, I15 | Essential (primary) hypertension; Secondary hypertension; |  |
|  |  |  |  |
| **Diabetes** | E10, E11, E13, E14 | Insulin-dependent diabetes mellitus; Non-insulin-dependent diabetes mellitus; Other specified diabetes mellitus;  Unspecified diabetes mellitus |  |
|  |  |  |  |
| **Dyslipidaemia** | E78.0, E78.1, E78.2, E78.4, E78.5, E78.6 | Pure hypercholesterolaemia;  Pure hypercholesterolaemia; Pure hyperglyceridaemia; Mixed hyperlipidaemia; Other hyperlipidaemia; Hyperlipidaemia, unspecified; Lipoprotein deficiency |  |
|  |  |  |  |
| **Angina** | I20, I21, I22 | Angina pectoris; Acute myocardial infarction; Subsequent myocardial infarction |  |
| **Ischaemic heart disease** | I24, I25 | Other acute ischaemic heart diseases; Chronic ischaemic heart disease |  |

The definitions are based on diagnoses (primary and secondary ICD10). ICD: international classification of disease.

**Supplementary Table 2:** Baseline characteristics of subjects used in test sets in model evaluation

|  | Female  (n = 14299) | Male  (n = 13372) |
| --- | --- | --- |
| Age | 64.0±7.6 | 65.3±7.9 |
| BMI (kg/m^2^) | 26.1±4.8 | 27.1±4.0 |
| VAT (L) | 2.62±1.5  (n = 2966) | 4.93±2.3  (n=2724) |
| Normal weight  (BMI<25 kg/m^2^) | 47.2% | 32.0% |
| Overweight  (25 kg/m^2^≤BMI<30 kg/m^2^) | 34.9% | 48.5% |
| Obese (BMI>30 kg/m^2^) | 17.9% | 19.5% |
| Hypertension | 10.5% | 17.7% |
| CHD | 2.5% | 7.5% |
| Diabetes | 1.9% | 4.0% |
| Dyslipidaemia | 4.1% | 9.2% |

Subjects from the fourth fold were excluded with only folds 1-3 used as test sets. BMI, body mass index; VAT, visceral adipose tissue; CHD, coronary heart disease. Continuous variables presented as mean+std.

**Supplementary Figure 1: Confusion matrices of binary classification (BMI>25kg/m^2^) and 3-class classification (BMI<25kg/m^2^, 25<BMI<30kg/m^2^, BMI>30kg/m2) for out-of-bag ECG test set.**


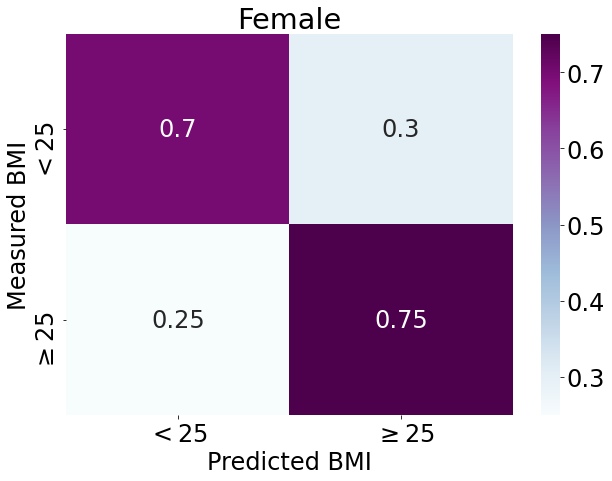

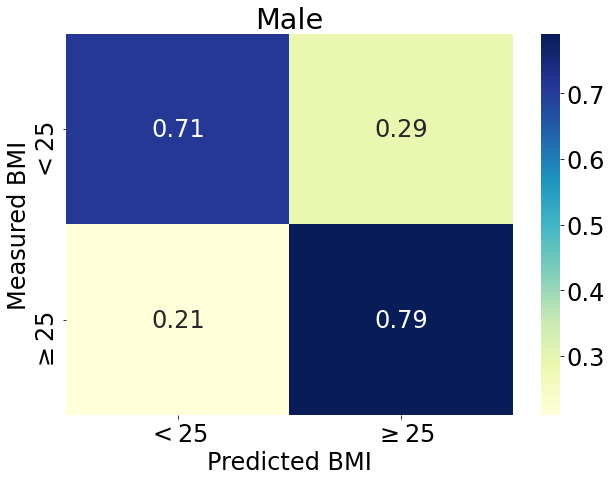

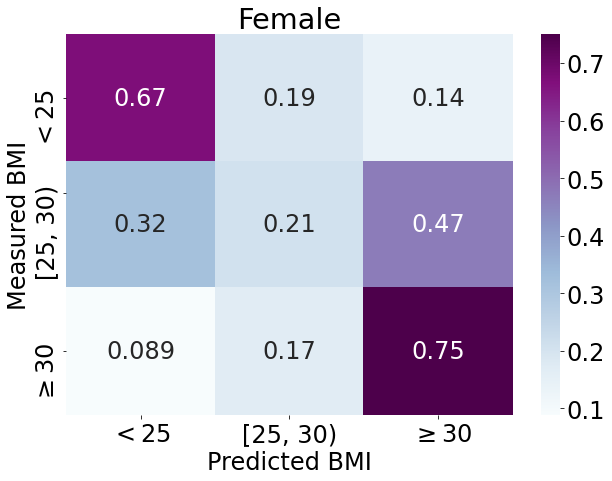

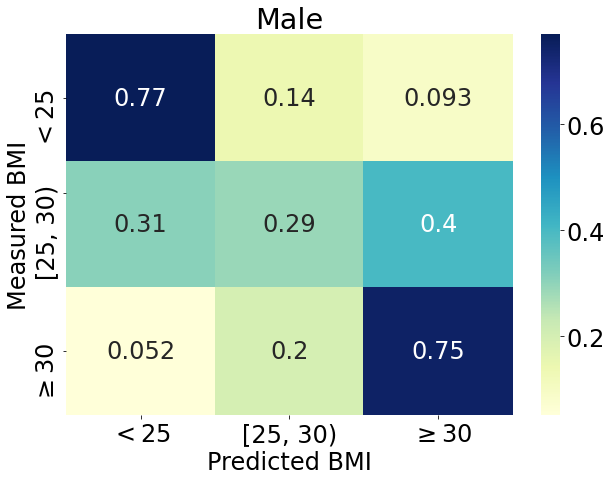


For the binary classification, the accuracies are 72% and 76% for females and males, respectively. For the 3-class classification, the accuracies are 52% and 54% for males and females, respectively.

**Supplementary Figure 2: Controlling for actual BMI, the greater the discrepancy between NN-predicted and actual BMI (ΔBMI), the greater the** Δ**VAT volume.**


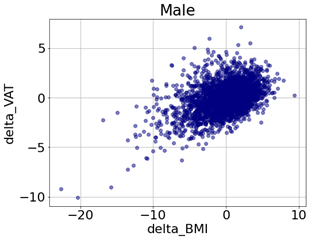


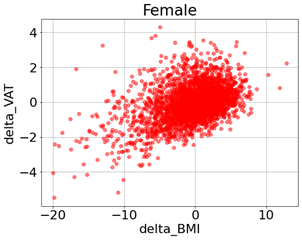


For both male and female participants, there is a positive association between the difference in NN-predicted and actual BMI (ΔBMI), and ΔVAT. This suggests that ΔBMI has captured information that would help to predict the portion of measured VAT not explained by conventionally measured BMI.

**References:**

1. Sudlow C, Gallacher J, Allen N et al. UK biobank: an open access resource for identifying the causes of a wide range of complex diseases of middle and old age. *PLoS Med*. 2015;12:e1001779.

2. Linge J, Borga M, West J et al. Body Composition Profiling in the UK Biobank Imaging Study. *Obesity (Silver Spring)*. 2018;26:1785-1795.

3. Banerjee I, Ling Y, Chen MC et al. Comparative effectiveness of convolutional neural network (CNN) and recurrent neural network (RNN) architectures for radiology text report classification. *Artif Intell Med*. 2019;97:79-88.

4. Hwang SJ, Mehta RR, Kim HJ, Johnson SC and Singh V. Sampling-free Uncertainty Estimation in Gated Recurrent Units with Applications to Normative Modeling in Neuroimaging. *Uncertain Artif Intell*. 2019;2019.

5. Liang G and Zheng L. A transfer learning method with deep residual network for pediatric pneumonia diagnosis. *Comput Methods Programs Biomed*. 2020;187:104964.

6. Weigend AS, Mangeas M and Srivastava AN. Nonlinear gated experts for time series: discovering regimes and avoiding overfitting. *Int J Neural Syst*. 1995;6:373-99.

7. Tomori S, Kadoya N, Takayama Y et al. A deep learning-based prediction model for gamma evaluation in patient-specific quality assurance. *Med Phys*. 2018.
